# *Elaeagnus angustifolia* Plant Extract Induces Apoptosis *via* P53 and Signal Transducer and Activator of Transcription 3 Signaling Pathways in Triple-Negative Breast Cancer Cells

**DOI:** 10.3389/fnut.2022.871667

**Published:** 2022-03-18

**Authors:** Arij Fouzat, Ola Jihad Hussein, Ishita Gupta, Halema F. Al-Farsi, Ashraf Khalil, Ala-Eddin Al Moustafa

**Affiliations:** ^1^College of Pharmacy, QU Health, Qatar University, Doha, Qatar; ^2^College of Medicine, QU Health, Qatar University, Doha, Qatar; ^3^Biomedical Research Centre, Qatar University, Doha, Qatar

**Keywords:** *Elaeagnus angustifolia*, triple-negative breast cancer, apoptosis, stat3, p53

## Abstract

*Elaeagnus angustifolia* (*EA*) is used as an alternative medicine in the Middle East to manage numerous human diseases. We recently reported that *EA* flower extract inhibits cell proliferation and invasion of human oral and HER2-positive breast cancer cells. Nevertheless, the outcome of *EA* extract on triple-negative breast cancer (TNBC) cells has not been explored yet. We herein investigate the effect of the aqueous *EA* extract (100 and 200 μl/ml) on two TNBC cell lines (MDA-MB-231 and MDA-MB-436) for 48 h and explore its underlying molecular pathways. Our data revealed that *EA* extract suppresses cell proliferation by approximately 50% and alters cell-cycle progression of these two cancer cell lines. Additionally, *EA* extract induces cell apoptosis by 40–50%, accompanied by the upregulation of pro-apoptotic markers (Bax and cleaved caspase-8) and downregulation of the anti-apoptotic marker, Bcl-2. Moreover, *EA* extract inhibits colony formation compared to their matched control. More significantly, the molecular pathway analysis of *EA*-treated cells revealed that *EA* extract enhances p53 expression, while inhibiting the expression of total and phosphorylated Signal Transducer and Activator Of Transcription 3 (STAT3) in both cell lines, suggesting p53 and STAT3 are the main key players behind the biological events provoked by the extract in TNBC cells. Our findings implicate that *EA* flower extract may possess an important potential as an anticancer drug against TNBC.

## Introduction

Breast cancer (BC) is the most prevalent cancer worldwide and is a commonly diagnosed malignancy in females comprising approximately one-third of all malignancies in women (1). Several genetic alterations, morphological characteristics, clinical outcome, and therapeutic interventions make BC a highly heterogeneous disease. BC is classified into four subtypes (Luminal A, Luminal B, HER-2 positive and triple-negative) to provide clinical utility along with sufficient prognostic and predictive power (2). Of the four subtypes, the triple-negative breast cancer (TNBC) lacks expression of estrogen and progesterone receptors (ER, PR) along with the absence or faint expression of the human epidermal growth factor receptor-2 (HER-2). TNBC accounts for around 12–20% of all breast cancer cases (3, 4); the incidence increases among pre-menopausal females and is more frequent in young women (4, 5). TN tumors are characterized by aggressive behavior with early metastasis to the central nervous system, bone, lung, and liver, along with a short response period to available therapies, poor prognosis and survival as compared to the other BC subtypes (6–8). Unlike ER and HER-2 positive BC, TNBCs are highly resistant to current therapies as they are insensitive to endocrine and molecular-targeted therapies; currently, systemic chemotherapy and surgery are the backbone of TNBC management (6, 9). In general, it is difficult to develop and evaluate novel agents against TNBC due to its extreme biologic heterogeneity. Nevertheless, triple-negative tumors become resistant to therapeutic modalities and can reoccur and develop metastatic abilities. Therefore, there is an urgent need to identify and develop novel potential therapeutic agents against TNBC which are effective with less undesired side effects.

Several complementary and alternative medicines (CAMs) in addition to chemotherapy drugs are largely inspired by nature, mainly plant based phytochemicals are used as a natural source for medical treatments (10, 11). Dietary phytochemicals are naturally occurring bioactive compounds that have the potential to be a competitive alternative for cancer treatment due to their efficacious and protective properties (10, 12). *Elaeagnus angustifolia* (*EA*), is one such a medicinal plant that has been used for centuries in folklore medicine in different parts of the world, especially in the Middle East region (13–15). *EA* is a rich source of vitamins, proteins, calcium, magnesium, potassium, and iron; hence, different parts of *EA*, either fresh or dried are consumed (16, 17). In fact, *EA* is used to treat multiple diseases like asthma, osteoporosis, and rheumatoid arthritis due to their antioxidant, anti-inflammatory, antimicrobial and anticancer properties (16, 18). In this context, it is important to highlight that the bioactive compounds in *EA* like flavonoid, lignanoid and bezenoid can have anti-tumor properties (19–21). However, data exploring the anti-cancer role of *EA* are limited to a few studies. Our group previously reported that *EA* extract suppresses cell invasion of human oral cancer cells *via* the Erk1/Erk2 signaling pathways (22). Another study revealed that hydroalcoholic *EA* flower extract significantly represses angiogenesis, indicating its potential as an anti-cancer drug (23). Our recent investigation on HER2-positive human breast cancer cells pointed out that *EA* aqueous extract significantly inhibits cell proliferation and provokes apoptosis by suppressing both, HER2 and JNK activation (24). However, there are no studies describing the outcome of *EA* extract against TNBC and its underlying molecular mechanisms. Therefore, we herein aimed to explore the potential therapeutic and anti-tumor characteristics of *EA* flower extract on TNBC cells and its mechanism.

In this investigation, we examined the effect of *EA* extract on cell proliferation, cell-cycle progression, apoptosis, and colony formation in two human TNBC cell lines. Our study revealed that EA induced dramatic cell apoptosis in TNBC cells, which occurs *via* p53 and Stat signaling pathways.

## Materials and Methods

### Plant Material Collection and Extraction

*Elaeagnus angustifolia* flowers were collected from Montreal, Quebec, Canada in June, and the aqueous extract was prepared as described previously by our group (24). Briefly, the flowers were dried and stored in the dark at room temperature. For extract preparation, 3 grams of dried *EA* flowers were boiled in 100 ml of autoclaved distilled water at 100°C for 15 min and stirred continuously. Following boiling, the *EA* flower extract solution was filtered using a sterile filter unit (0.45 μm pore size) and stored at 4°C for future experimental use. Dilutions of *EA* extracts were prepared in cell culture media and for each set of experiments the extract was freshly prepared.

### Cell Culture

Two different human TNBC cell lines (MDA-MB-231 and MDA-MB-436) and the non-tumorigenic epithelial cell line (MCF 10A) were commercially obtained from the American Type Culture Collection (ATCC) (Rockville, MD, United States). The cell lines were cultured in Dulbecco’s Modified Eagle’s Media-high glucose (DMEM, Sigma-Aldrich, St. Louis, MO, United States) supplemented with 10% fetal bovine serum (FBS, Gibco, Life Technologies, Massachusetts, MA, United States), 10 mM non-essential amino acids (Gibco, Life Technologies, Massachusetts, MA, United States), 0.5 mM sodium pyruvate (Gibco, Life Technologies, Massachusetts, MA, United States), 2.5 mM L-glutamine (Gibco, Life Technologies, Massachusetts, MA, United States), 1% antibiotic (penicillin-streptomycin, Gibco, Life Technologies, Massachusetts, MA, United States) at 37*^o^*C with 5% CO_2_ and 85% humidity. All experiments were carried out when the cells had attained 70–80% confluence.

### Cell Viability

The TNBC cell lines (MDA-MB-231 and MDA-MB-436) and the non-malignant breast epithelial cell line (MCF 10A) were seeded in 96-well plates with a density of 1 × 10^4^ cells per well. The cells were grown in DMEM medium as described above. MDA-MB-231, MDA-MB-436 and MCF 10A cells were treated with different concentrations (25, 50, 75, 100, 150, and 200 μl/ml) of *EA* extract solution for 48 h. Untreated cells were used as the control and were cultured in 100 μl of media. AlamarBlue™ cell viability assay (Invitrogen, Thermo Fisher Scientific, Waltham, MA, United States) was used to determine the effect of *EA* extract on cell proliferation according to the manufacturer’s protocol. After incubating the cells for 4 h with the dye, the shift in fluorescence was measured at two wavelengths, excitation wavelength at 570 nm and emission wavelength at 590 nm using a fluorescent plate reader (Infinite M200, Tecan, Grödig, Austria). The percentage of cell viability was calculated based on the fluorescence of treated cells to untreated cells.

### Cell Morphology Analysis

The two TNBC cell lines (MDA-MB-231 and MDA-MB-436) and MCF 10A cells were seeded in 6-well plates (2.5 × 10^5^ cells/well) for 24 h. The old medium was then discarded, and cells were treated with the half-maximal inhibitory concentration (IC_50_) of *EA* extract (100 and 200 μl/ml) for 48 h. Cell morphology was examined using a light microscope with 10X magnification after 24 and 48 h. The changes of cell morphology under the effect of *EA* treatment were analyzed by capturing images of the cells with the Leica DFC550 digital camera (Leica Microsystems, Wetzlar, Germany) at 12.5 Megapixel resolution.

### Cell Cycle Analysis

For cell cycle analysis, a total of 1 × 10^6^ cells of the TNBC cells were seeded in 100 mm Petri dishes for 24 h. To synchronize the cells into the G0 phase, seeded cells were starved overnight with serum-free DMEM medium. Synchronized cells were treated with *EA* aqueous extract (100 and 200 μl/ml) for 48 h. Post treatment, the cells were fixed with ice-cold 70% ethanol. The DNA was then stained using the FXCycle PI/RNase staining solution (Invitrogen, Thermo Fisher Scientific) at 50 μg/ml according to the manufacturer’s recommendations. Cells in the G0/G1, S, and G2/M phases were quantified using the Flow Jo software.

### Annexin V Apoptosis Assay

Cellular apoptosis was assessed by the ApoScreen^®^ Annexin V- fluorescein isothiocyanate (FITC)/7-amino-actinomycin D (7-AAD) Apoptosis Kit (SouthernBiotech, United States) as per the manufacturer’s protocol. Briefly, TNBC cells were seeded in 100 mm Petri dishes at a density of 1 × 10^6^ cells/dish and incubated overnight. The following day, cells were treated with *EA* extract at a concentration of 100 and 200 μl/ml for 48 h. Adherent and floating cells were harvested by trypsinization, washed twice with cold PBS, and resuspended in Annexin Binding Buffer, then stained with conjugated Annexin V-FITC, PI or both stains for 15 min. After staining, samples were analyzed by BD FACSAria™ II Flow Cytometer and Flow Jo software. The cell population, excluding debris, was gated with forward scatter (FSC-A) and side scatter (SSC-A). Doublets were excluded using FSC-height and FSC-Width plot and singlet cells were then presented as dot plots of FITC-A (annexinV) against PerCP-Cy5.5-A (7-AAD). Quad Gates were used to calculate the percentage of viable cells (annexin V low, 7-AAD low), early pro-apoptotic cells (annexin V high, 7-AAD low) and late apoptosis/necrotic cells (annexin V high, 7-AAD high). Data were presented as density plots of Annexin V-FITC and 7-AAD staining.

### Soft Agar Colony Formation Assay

To investigate the ability of TNBC cells to grow in an anchorage-independent manner, soft agar colony formation assay was performed as previously described by our group (25). Briefly, TNBC cells were seeded in 6-well plates (1 × 10^4^ cells/well) with/without 100 and 200 μl/ml of *EA* extract (treated/control, respectively) placed in DMEM medium containing 0.3% agar and plated over a layer of DMEM medium with 10% FBS and containing 0.4% agar. The growing colonies were examined every 2 days for a period of 3 weeks. Colonies in each well were counted under a light microscope, Leica SP8 UV/Visible Laser confocal microscope (Leica Microsystems, Wetzlar, Germany), in five predetermined fields.

### Western Blot Analysis

The immunoblotting analysis was performed as previously described by our group (20, 25). Briefly, a total of 1 × 10^6^ cells of TNBC cells (MDA-MB-231 and MDA-MB-436) were seeded in Petri dishes and treated with the aqueous extract of *EA* flowers (100 and 200 μl/ml) for 48 h. Protein lysates were extracted from the control and treated cells. Then, equal amounts of protein were run on 10% polyacrylamide gels and transferred onto PVDF membranes. The membranes were incubated with the following primary antibodies overnight; mouse anti-Bax (ThermoFisher Scientific: MA5-14003), mouse anti-Bcl-2 (Abcam: abID# ab692), mouse anti-cleaved caspase-8 (Cell Signaling Technology, CST: mAb #9748), rabbit anti-p53 (Cell Signaling Technology, CST: mAb #2527), mouse anti-Signal Transducer and Activator Of Transcription 3 (STAT3) (Cell Signaling Technology, CST: mAb #9139) and rabbit anti-phosphorylated-STAT3 (Cell Signaling Technology, CST: mAb #9131). To confirm the equal loading of proteins, PVDF membranes were re-probed with rabbit anti-GAPDH (Abcam: abID# ab9485). Post primary antibody staining, membranes were probed with an anti-rabbit IgG-HRP (Cell Signaling Technology, CST:7074S) or anti-mouse IgG-HRP (Cell Signaling Technology, CST: 7076S) secondary antibody.

Chemiluminescence ECL-Western blotting substrate kit (Pierce Biotechnology, Waltham, MA, United States) was used to detect the immunoreactivity as described by the manufacturer. Quantification was carried out by the ImageJ software and the bands’ intensities was normalized to GAPDH to determine the relative protein expression in each cell line.

### Statistical Analysis

All experiments were analyzed using the IBM Statistical Package for the Social Sciences (SPSS) version 27 software. One-way ANOVA test was performed to analyze the difference between *EA* treated and untreated cells, followed by Tukey’s multiple comparisons test. The data was presented as mean ± S.E.M. from three independent experiments (*n* = 3). *P-values* < 0.05 were considered significant.

## Results

**FIGURE 1:**
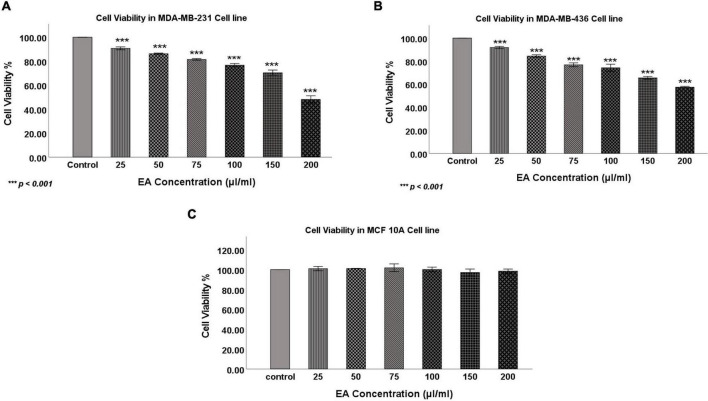
The effect of EA flowers extract at different concentrations on cell viability of the TNBC cell lines **(A)** MDA-MB-231, **(B)** MDA-MB-436 and the **(C)** non-tumorigenic cell line, MCF 10A after 48 h of treatment. Data shows an inverse relation between treatment concentrations and cell viability in both TNBC cell lines, whereas MCF 10A cells’ viability was not affected. Data are expressed as a percent of growth relative to control (mean ± SEM; *n* = 3). One-way ANOVA followed by Tukey’s *post hoc* test was used to compare the treatment groups and results were considered statistically significant when *p* < 0.05 in comparison with the control. ***p < 0.001.

To determine the anticancer activity of the aqueous extract of EA flowers on the TNBC, two cell lines, MDA-MB-231 and MDA-MB-436, were treated with EA extract at different concentrations (0, 25, 50, 75, 100, 150, and 200 μl/ml) for 48 h. Our data showed that EA extract significantly inhibits the proliferation of both cell lines in a dose-dependent manner (Figures 1A,B). More specifically, treatment with 100 and 200 μl/ml inhibited the proliferation of MDA-MB-231 cells by 30 and 55%, respectively, while for MDA-MB-436 cells, proliferation was reduced by 40 and 50%, respectively. Notably, we found that 100 and 200 μl/ml concentrations of the EA extract has no significant effect on cell viability of the non-tumorigenic epithelial cell line, MCF 10A (Figure 1C) and hence concentrations of 100 and 200 μl/ml were selected for further investigations in both TNBC cell lines.

**FIGURE 2:**
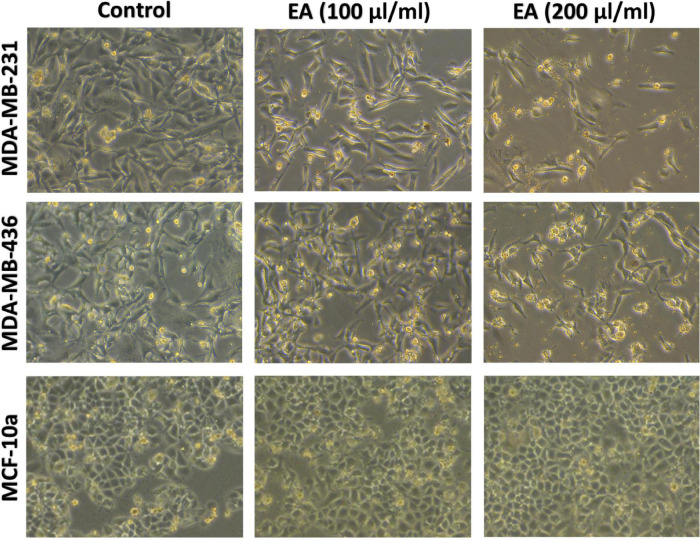
Effect of EA extract on cell morphology of MDA-MB-231, MDA-MB-436 and MCF 10A. We note that treatment for 48 h with 100 and 200 μl/ml of *EA* extract inhibits cell proliferation and induces death in both cell lines, in comparison with untreated (control) which show no cytotoxic effect and display a healthy fibroblast-like phenotype cell. Images were taken at 10x magnification.

Next, we examined the cell morphology of MDA-MB-231, MDA-MB-436 and MCF 10A under the effect of 100 and 200 μl/ml of *EA* extract, using phase-contrast microscopy. As shown in Figure 2, untreated TNBC cells (control), MDA-MB-231 and MDA-MB-436, were highly confluent and displayed fibroblast-like phenotype. However, following treatment with 100 μl/ml of *EA* extract, TNBC cells started losing their shape and underwent several morphological changes including deformation, loss of membrane integrity, contact inhibition, cell shrinkage, and formation of apoptotic bodies. At higher concentration of *EA* (200 μl/ml), the morphological changes become more evident with a profound increase in detaching cells and reduction in the number of viable cells, indicating cell death in TNBC. Contrary, this effect was not observed in the non-tumorigenic cell line MCF 10A, as shown in Figure 2.

**FIGURE 3:**
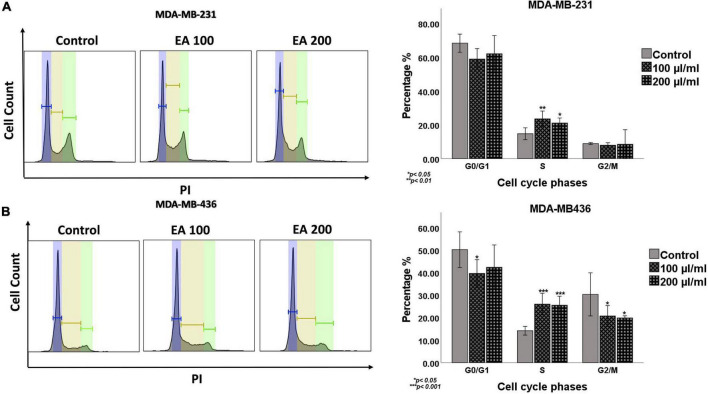
Cell cycle flow cytometry analysis of the TNBC cell lines, **(A)** MDA-MB-231 and **(B)** MDA-MB-436. Cells were treated with 100 and 200 μl/ml of *EA* extract for 48 h followed by staining with propidium iodide (PI). Representative DNA content histogram showing the phases of sub G0, G0\G1, S, and G2/M on tested cell lines upon treatment with *EA* extract compared to their matched control. The cell cycle histogram results reveal that EA extract at these concentrations induce a significant cell cycle arrest at the S phase, with a notable decrease in G0/G1 and G2/M phases. Quantification is represented as the mean ± SEM (*n* = 3). One-way ANOVA followed by Tukey’s *post hoc* test was used to compare the treatment groups and statistical significance was indicated when *p* < 0.05. *p < 0.05, **p < 0.01, and ***p < 0.001.

Subsequently, the effect of *EA* on cell cycle progression of TNBC cells was investigated using flow cytometric tool. As shown in Figure 3, our data revealed that *EA* extract at 100 and 200 μl/ml concentrations induce a significant cell cycle arrest at the S phase, with a notable decrease in G0/G1 and G2/M phases of both TNBC cell lines, indicating inhibition of cell-cycle progression under the effect of *EA* extract.

**FIGURE 4:**
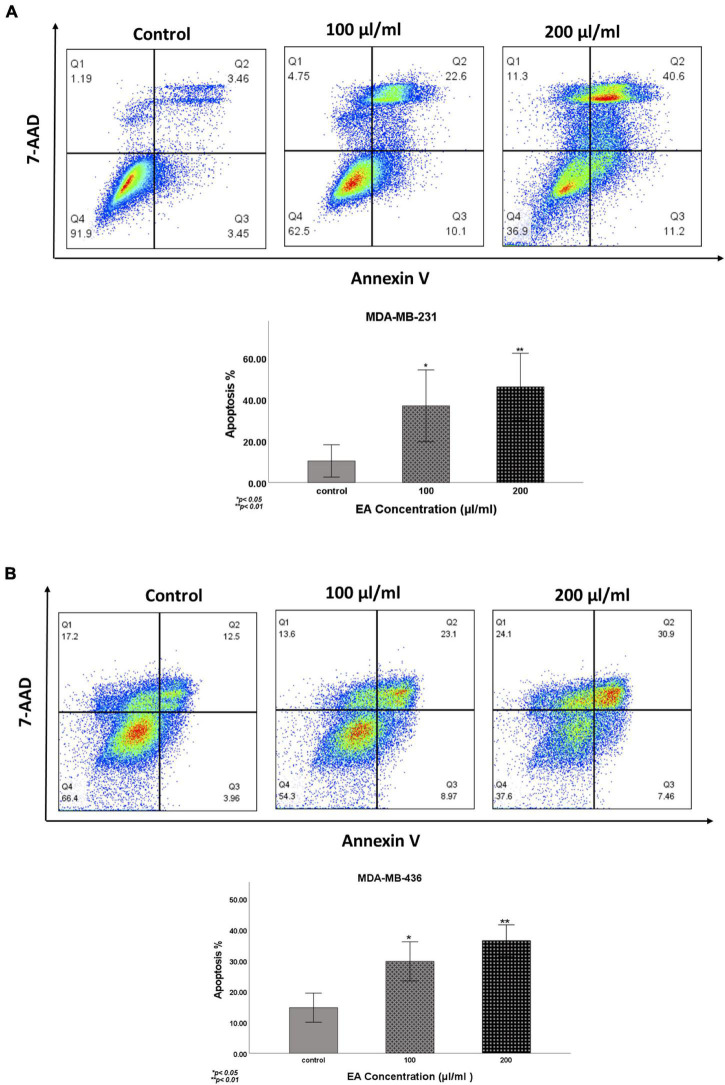
Induction of apoptosis by EA extract in **(A)** MDA-MB-231 and **(B)** MDA-MB-436 cells, as determined by Annexin V-FITC and 7-AAD apoptosis assay. Cells were treated with 100 and 200 μl/ml of *EA* extract for 48 h. Data are presented as Mean ± SEM (*N* = 3). Results were analyzed using One-way ANOVA followed by Dunnett’s *post hoc* test. *p* < 0.05 was considered for statistical significance. *p < 0.05 and **p < 0.01.

To further assess the effect of *EA* extract on cell apoptosis, we performed Annexin V-FITC/7-AAD assay. Our data reveals that *EA* extract significantly induces early and late apoptosis in a dose-dependent manner in both TNBC cell lines (Figure 4).

**FIGURE 5:**
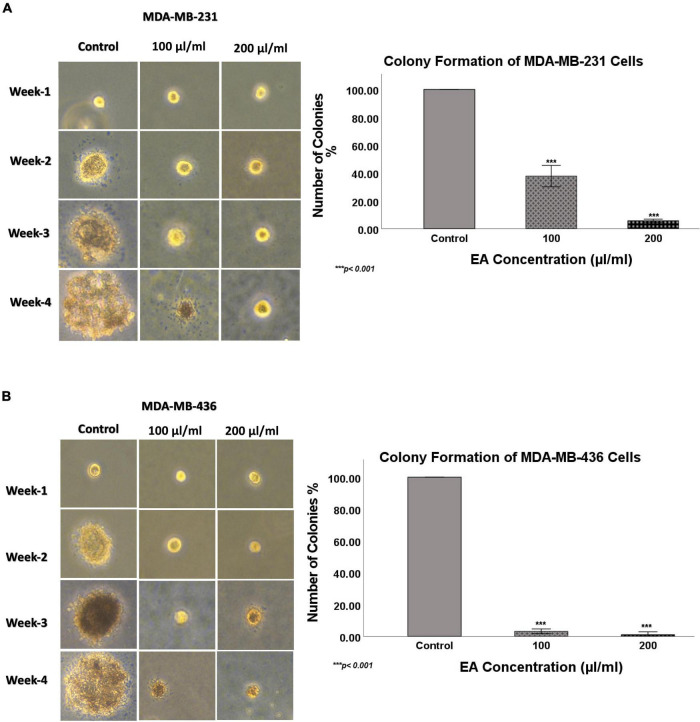
The effect of EA extract on colony formation, in soft agar, in human TNBC cell lines **(A)** MDA-MB-231 and **(B)** MDA-MB-436. *EA* extract inhibits colony formation of MDA-MB-231 and MDA-MB-436, in comparison with their matched control cells. Images were taken at 10x magnification. The colonies were counted manually and expressed as percentage of treatment relative to the control (mean ± SEM; *n* = 3). ***p < 0.001.

Next, we explored the effect of *EA* extract (100 and 200 μl/ml concentrations) on the colony formation of TNBC cell lines in soft agar over a period of 4 weeks. Our data showed a significant decrease in the number of colonies for both TNBC cell lines under the effect of *EA* extract compared to the control, as shown in Figure 5. *EA* extract inhibited colony formation of MDA-MB-231 by 62 and 94% at 100 and 200 μl/ml of *EA* extract, respectively. On the other hand, the number of colonies formed in MDA-MB-436 cells was reduced by 96 and 99% at 100 and 200 μl/ml of *EA* extract, correspondingly, in comparison to the control. These results prove loss of colony-forming ability in both TNBC cell lines upon treatment with *EA* extract, which may reflect the ability to inhibit tumorigenesis *in vivo*.

**FIGURE 6:**
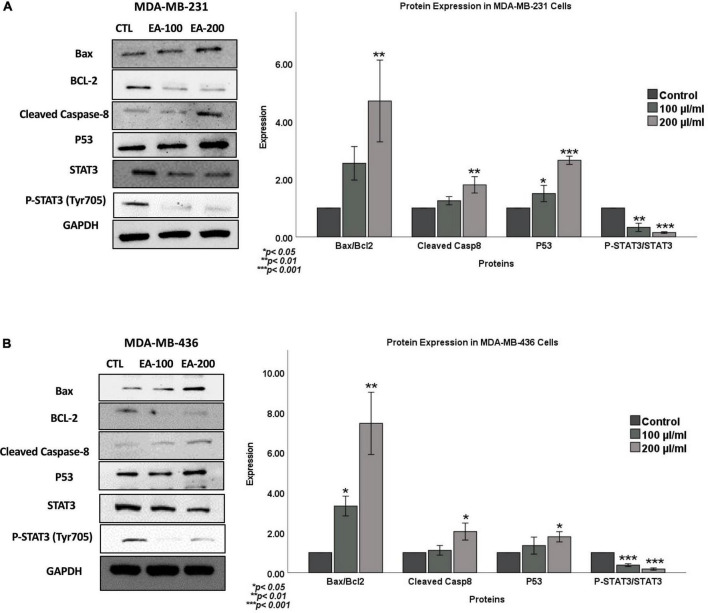
Outcome of EA extract on the expression patterns of Bax, BCL-2, Caspase-8, P53 and STAT3 in **(A)** MDA-MB-231 and **(B)** MDA-MB-436 cell lines. We note that *EA* extract induces an overexpression of the pro-apoptotic markers (Bax and Caspase-8) in comparison with their control, while anti-apoptotic marker Bcl-2 is inhibited. Furthermore, *EA* plant extract enhances the expression of p53 while inhibits phosphorylation of STAT3. GAPDH served as a control in this assay. Cells were treated with 100 and 200 μl/ml of *EA* extract for 48 h as explained in the materials and methods section. Values were corrected for the expression of the housekeeping protein GAPDH and presented as fold change of control. Data were analyzed using One-way ANOVA followed by Dunnett’s *post hoc* test. *P-value < *0.05* was considered for statistical significance. *p < 0.05, **p < 0.01, and ***p < 0.001. Data are presented as a percentage of treatment relative to the control (Mean ± SEM; *n* = 3).

To further confirm the role of *EA* extract on apoptosis, we examined the expression patterns of key markers of apoptosis in TNBC cells (MDA-MB-231 and MDA-MB-436) following 48 h of treatment with 100 and 200 μl/ml of *EA* extract using western blot analysis. Our data revealed a significant increase in the expression of the pro-apoptotic markers (Bax and cleaved caspase-8) in *EA*-treated cells compared to control, as shown in Figure 6. On the other hand, the expression of the anti-apoptotic protein (Bcl-2) was reduced in both cell lines. In parallel, Bax/Bcl-2 ratio was profoundly increased in cells treated with *EA* extract, as seen in Figure 6. Taken together, these findings provide evidence that *EA* extract can induce apoptosis in TNBC cells, which is associated with the deregulation of Bcl-2/Bax/caspase signaling pathway.

Regarding the underlying molecular pathways of *EA* extract on cell viability, apoptosis and inhibition of colony formation of TNBC cells, we postulate that p53 and STAT3, which are commonly altered in TNBCs (26, 27), could play a vital role in the regulation of these biological events under the effect of *EA* extract. Thus, we evaluated the expression pattern of p53, STAT3 and p-STAT-3 in MDA-MB-231 and MDA-MB-436 cell lines exposed to *EA* extract in comparison with their matched (untreated cells) controls. We found that treatment with *EA* extract stimulates the expression pattern of p53 whereas, *EA* extract inhibits the expression and phosphorylation of STAT3 in both cell lines in comparison to their control cells.

## Discussion

Natural products have continuously proven to be an important and rich source of therapies for a variety of human disorders, including cancer (10, 11). In the present study, we investigated for the first time the effect of *EA* flower extract in TNBC cell lines (MDA-MB-231 and MDA-MB-436) with regards to cell proliferation, morphological changes, cell cycle and apoptosis as well as the underlying molecular pathways. We herein report that *EA* extract can inhibit cell proliferation, alter the normal morphology and deregulate cell cycle progression in addition to the induction of cell apoptosis of TNBC cell lines while having a minimal effect on the growth of non-tumorigenic epithelial cell line, MCF 10A. Moreover, we noted that the aqueous extract of *EA* flower can inhibit colony formation of TNBC cells, which correlates with *in vivo* tumor inhibition. In our laboratory, we also found that *EA* extract significantly enhanced the survival rate of both, wild-type and K-RAS mutant *Drosophila melanogaster* flies, which are prone to developing colorectal cancer. This *in vivo* data show clearly that *EA* extract can block colorectal cancer growth, indicating anti-cancer role of *EA* extract (in preparation).

*Elaeagnus angustifolia* plant is traditionally used for centuries as an analgesic, antipyretic, anti-inflammatory, antioxidant, and diuretic herbal medicine (13–15, 28). Moreover, several bioactive compounds are identified in *EA*, such as phenolic acids and flavonoids, which are thought to play a role in preventing cancer development and progression (18, 20). Studies have demonstrated that these compounds can regulate multiple cellular processes such as DNA repair, cell cycle progression, induction of apoptosis and cell signaling cascades (29, 30). Recently, there has been considerable interest in their potential therapeutic utility as chemo-preventive and/or anticancer agents. Nevertheless, to the best of our knowledge, the effect of this medicinal plant on TNBC has not been previously explored. Therefore, in this study we evaluated the cytotoxic potential of *EA* in TNBC. Particularly, *EA* flowers, which is commonly consumed as tea beverage in several cultures and especially the middle east, were extracted by decoction and tested on TNBC cell lines to examine the outcome of *EA* flowers dietary intake on TNBC (31).

On the other hand, uncontrolled cell proliferation and evasion of apoptosis are well-recognized hallmarks of cancer and vital components of treatment resistance; hence, targeting associated deregulated pathways is considered as the main cancer therapeutics tool (32, 33). Our data reveals that *EA* plant extract inhibits cell proliferation of TNBC cells, meanwhile it provokes cell apoptosis in our TNBC cell models, which is accompanied by upregulation of proapoptotic proteins (Bax, cleaved caspase-8) and downregulation of anti-apoptotic protein (Bcl-2). Interestingly, our data of non-tumorigenic cells, show that *EA* extract has a very minimal or no toxic effect in the control cell line, MCF10, suggesting a selective cytotoxic activity in breast cancer cells, which is a favorable property in the development of anticancer agents. These findings are in concordance with our recent reports on the anticancer potential of *EA* against human oral and HER2 + breast cancer cells (22, 24). Interestingly, the underlying molecular mechanisms preventing tumor progression in oral and HER2 + cells targeted different pathways. In oral cancer cells, EA inhibited angiogenesis and cell invasion *via* Erk1/Erk2 signaling pathways (22). On the other hand, in HER2 + cells, we found *EA* to inhibit epithelial-mesenchymal transition and provoke apoptosis *via* HER2 inactivation and JNK pathway (24).

Analysis of the underlying molecular pathway reveals that *EA* extract provokes apoptosis, which might be at least partially mediated through the upregulation of the tumor suppressor gene, p53 and the accompanied suppression of STAT3 signaling. P53 controls the transcription of proapoptotic members of the Bcl-2 family, such as Bax, Bid, Noxa, and Puma, resulting in apoptosis induction (34, 35). We thus analyzed the expression of the tumor suppressor gene p53. Notably, we observed that *EA* extract significantly upregulates the expression of p53 and its downstream signaling (Bax). On the other hand, STAT3 possesses oncogenic potential and contributes to cancer cell proliferation, anti-apoptosis, migration, invasion, immune suppression, stemness and resistance to chemotherapy (26, 36, 37). Recent evidence has demonstrated that STAT3 is overexpressed and constitutively activated in TNBC, which is associated with the initiation, progression, and metastasis of TNBC (26, 38). The oncogenic potential of STAT3 is triggered by its phosphorylation on Tyr705, which results in homo- or hetero-dimerization of STAT3 followed by nuclear translocation, binding STAT3 to specific DNA response elements and activating target genes (39–41). STAT3 regulates the expression of several proteins, including proliferation regulatory proteins (cyclin D1 and survivin) and anti-apoptotic proteins (Bcl-2 and Bcl-xl) (41–43). Interestingly, our data demonstrate that EA extract inhibits the expression of STAT3, phospho-STAT3, as well as their target genes, including the anti-apoptotic protein Bcl-2. These findings were correlated with increased apoptosis as indicated by the accumulation of caspase-3 and caspase-8, leading to increased numbers of apoptotic cells as evidenced by the Annexin-V staining. Taken together, these results suggest that EA extract induced apoptosis could be mediated through STAT3 dependent mechanism.

Interestingly, given that STAT3 and p53 have opposing roles, where p53 promotes the apoptotic pathway and activation of STAT3 upregulates survival signals; it has been shown that p53 and STAT3 engage in an interplay where they negatively regulate each other (44, 45). Several studies have shown that blocking STAT3 activity in cancer cells promotes the expression of p53, resulting in p53-mediated cell apoptosis (45, 46). More specifically, Lin et al. reported that expression of wild-type p53 but not mutant p53 significantly reduces tyrosine phosphorylation of STAT3 and inhibits STAT3 DNA binding in DU145 prostate cancer cell line (47). Moreover, several studies have shown an association between STAT3 activation and p53 mutations with therapy resistance in cancer (45, 48, 49). Therefore, dual targeting of p53 and STAT3 could be a promising approach to overcome therapy resistance. Collectively, our data reveal that *EA* could simultaneously upregulate p53 while downregulating STAT3 signaling, suggesting that it may serve as a potential effective treatment in TNBC. Future work includes extracting and analyzing the phytochemical components of the *EA* extract and testing the anti-cancer efficacy of these phytochemicals.

## Conclusion

Our present study establishes for the first time the anticancer potential of *EA* flower extract against TNBC and its molecular signaling pathway. Moreover, we herein report EA extract provokes cell apoptosis in TNBC cells; this is accompanied by the deregulation of proapoptotic and anti-apoptotic genes. Additionally, our data highlight the role of p53 and STAT3 pathways as a potential target for TNBC therapy *via* natural products. Collectively, this study demonstrates the role of *EA* as a promising therapeutic candidate for breast cancer management, particularly the TNBC subtype.

## Data Availability Statement

The original contributions presented in the study are included in the article/supplementary material, further inquiries can be directed to the corresponding author.

## Author Contributions

A-EA: conceptualization. AF and OH: methodology, validation, and data curation. A-EA, HA-F, and AK: resources, writing—review and editing, and supervision. AF, OH, and IG: writing—original draft preparation. AK and HA-F: funding acquisition. All authors have read and agreed to the published version of the manuscript.

## Conflict of Interest

The authors declare that the research was conducted in the absence of any commercial or financial relationships that could be construed as a potential conflict of interest.

## Publisher’s Note

All claims expressed in this article are solely those of the authors and do not necessarily represent those of their affiliated organizations, or those of the publisher, the editors and the reviewers. Any product that may be evaluated in this article, or claim that may be made by its manufacturer, is not guaranteed or endorsed by the publisher.

## References

[1] Sung H Ferlay J Siegel RL Laversanne M Soerjomataram I Jemal A Global cancer statistics 2020: GLOBOCAN estimates of incidence and mortality worldwide for 36 cancers in 185 countries. *CAA Cancer J Clin.* (2021) 71:209–49. 10.3322/caac.21660 33538338

[2] Perou CM Sørlie T Eisen MB van de Rijn M Jeffrey SS Rees CA Molecular portraits of human breast tumours. *Nature.* (2000) 406:747–52. 10.1038/35021093 10963602

[3] Wahba HA El-Hadaad HA. Current approaches in treatment of triple-negative breast cancer. *Cancer Biol Med.* (2015) 12:106–16. 10.7497/j.issn.2095-3941.2015.0030 26175926 PMC4493381

[4] Anders CK Carey LA. Biology, metastatic patterns, and treatment of patients with triple-negative breast cancer. *Clin Breast Cancer.* (2009) 9(Suppl. 2):S73–81. 10.3816/CBC.2009.s.008 19596646 PMC2919761

[5] Millikan RC Newman B Tse CK Moorman PG Conway K Dressler LG Epidemiology of basal-like breast cancer. *Breast Cancer Res Treat.* (2008) 109:123–39. 10.1007/s10549-007-9632-6 17578664 PMC2443103

[6] Nedeljković M Damjanović A. Mechanisms of chemotherapy resistance in triple-negative breast cancer-how we can rise to the challenge. *Cells.* (2019) 8:957. 10.3390/cells8090957 31443516 PMC6770896

[7] Dent R Trudeau M Pritchard KI Hanna WM Kahn HK Sawka CA Triple-negative breast cancer: clinical features and patterns of recurrence. *Clin Cancer Res.* (2007) 13(15 Pt 1):4429–34. 10.1158/1078-0432.Ccr-06-3045 17671126

[8] Kennecke H Yerushalmi R Woods R Cheang MC Voduc D Speers CH Metastatic behavior of breast cancer subtypes. *J Clin Oncol.* (2010) 28:3271–7. 10.1200/jco.2009.25.9820 20498394

[9] Lebert JM Lester R Powell E Seal M McCarthy J. Advances in the systemic treatment of triple-negative breast cancer. *Curr Oncol.* (2018) 25(Suppl. 1):S142–50. 10.3747/co.25.3954 29910657 PMC6001760

[10] Atanasov AG Waltenberger B Pferschy-Wenzig EM Linder T Wawrosch C Uhrin P Discovery and resupply of pharmacologically active plant-derived natural products: a review. *Biotechnol Adv.* (2015) 33:1582–614. 10.1016/j.biotechadv.2015.08.001 26281720 PMC4748402

[11] Atanasov AG Zotchev SB Dirsch VM Orhan IE Banach M Rollinger JM Natural products in drug discovery: advances and opportunities. *Nat Rev Drug Discov.* (2021) 20:200–16. 10.1038/s41573-020-00114-z 33510482 PMC7841765

[12] Harvey AL Edrada-Ebel R Quinn RJ. The re-emergence of natural products for drug discovery in the genomics era. *Nat Rev Drug Discov.* (2015) 14:111–29. 10.1038/nrd4510 25614221

[13] Amiri Tehranizadeh Z Baratian A Hosseinzadeh H. Russian olive (*Elaeagnus angustifolia*) as a herbal healer. *Bioimpacts.* (2016) 6:155–67. 10.15171/bi.2016.22 27853679 PMC5108988

[14] Ahmadiani A Hosseiny J Semnanian S Javan M Saeedi F Kamalinejad M Antinociceptive and anti-inflammatory effects of *Elaeagnus angustifolia* fruit extract. *J Ethnopharmacol.* (2000) 72:287–92. 10.1016/s0378-8741(00)00222-1 10967484

[15] Gürbüz I Ustün O Yesilada E Sezik E Kutsal O. Anti-ulcerogenic activity of some plants used as folk remedy in Turkey. *J Ethnopharmacol.* (2003) 88:93–7. 10.1016/s0378-8741(03)00174-0 12902057

[16] Hamidpour R Hamidpour S Hamidpour M Shahlari M Sohraby M Shahlari N Russian olive (*Elaeagnus angustifolia* L.): from a variety of traditional medicinal applications to its novel roles as active antioxidant, anti-inflammatory, anti-mutagenic and analgesic agent. *J Tradit Complement Med.* (2017) 7:24–9. 10.1016/j.jtcme.2015.09.004 28053884 PMC5198788

[17] Boudraa S Hambaba L Zidani S Boudraa H. Mineral and vitamin composition of fruits of five underexploited species in Algeria: *Celtis australis* L., *Crataegus azarolus* L., *Crataegus monogyna* Jacq., *Elaeagnus angustifolia* L. and *Zizyphus lotus* L. *Int J Trop Subtrop Horticult.* (2010) 65:75–84.

[18] Saboonchian F Jamei R Hosseini Sarghein S. Phenolic and flavonoid content of *Elaeagnus angustifolia* L. (leaf and flower). *Avicenna J Phytomed.* (2014) 4:231–8. 25068137 PMC4110780

[19] Ayaz FA Bertoft E. Sugar and phenolic acid composition of stored commercial oleaster fruits. *J Food Composit Anal.* (2001) 14:505–11. 10.1006/jfca.2001.1004

[20] Carradori S Cairone F Garzoli S Fabrizi G Iazzetti A Giusti AM Phytocomplex characterization and biological evaluation of powdered fruits and leaves from *Elaeagnus angustifolia*. *Molecules.* (2020) 25:2021. 10.3390/molecules25092021 32357533 PMC7248930

[21] Abizov EA Tolkachev ON Mal’tsev SD Abizova EV. Composition of biologically active substances isolated from the fruits of Russian olive (*Elaeagnus angustifolia*) introduced in the European part of Russia. *Pharm Chem J.* (2008) 42:696–8. 10.1007/s11094-009-0203-5

[22] Saleh AI Mohamed I Mohamed AA Abdelkader M Yalcin HC Aboulkassim T *Elaeagnus angustifolia* plant extract inhibits angiogenesis and downgrades cell invasion of human oral cancer cells via Erk1/Erk2 inactivation. *Nutr Cancer.* (2018) 70:297–305. 10.1080/01635581.2018.1412472 29300111

[23] Badrhadad A Piri KH Mansouri K. In vitro anti-angiogenic activity fractions from hydroalcoholic extract of *Elaeagnus angustifolia* L. flower and *Nepeta crispa* L. arial part. *J Med Plants Res.* (2012) 6:4633–9. 10.5897/jmpr11.1573

[24] Jabeen A Sharma A Gupta I Kheraldine H Vranic S Al Moustafa AE *Elaeagnus angustifolia* plant extract inhibits epithelial-mesenchymal transition and induces apoptosis via HER2 inactivation and JNK pathway in HER2-positive breast cancer cells. *Molecules.* (2020) 25:4240. 10.3390/molecules25184240 32947764 PMC7570883

[25] Kheraldine H Gupta I Alhussain H Jabeen A Cyprian FS Akhtar S Substantial cell apoptosis provoked by naked PAMAM dendrimers in HER2-positive human breast cancer via JNK and ERK1/ERK2 signalling pathways. *Comput Struct Biotechnol J.* (2021) 19:2881–90. 10.1016/j.csbj.2021.05.011 34093999 PMC8144105

[26] Qin J-J Yan L Zhang J Zhang W-D. STAT3 as a potential therapeutic target in triple negative breast cancer: a systematic review. *J Exp Clin Cancer Res.* (2019) 38:195. 10.1186/s13046-019-1206-z 31088482 PMC6518732

[27] Jin MS Park IA Kim JY Chung YR Im SA Lee KH New insight on the biological role of p53 protein as a tumor suppressor: re-evaluation of its clinical significance in triple-negative breast cancer. *Tumour Biol.* (2016) 37:11017–24. 10.1007/s13277-016-4990-5 26894602

[28] Farzaei MH Bahramsoltani R Abbasabadi Z Rahimi R. A comprehensive review on phytochemical and pharmacological aspects of *Elaeagnus angustifolia* L. *J Pharm Pharmacol.* (2015) 67:1467–80. 10.1111/jphp.12442 26076872

[29] Hui C Qi X Qianyong Z Xiaoli P Jundong Z Mantian M. Flavonoids, flavonoid subclasses and breast cancer risk: a meta-analysis of epidemiologic studies. *PLoS One.* (2013) 8:e54318. 10.1371/journal.pone.0054318 23349849 PMC3548848

[30] Zhang H-W Hu J-J Fu R-Q Liu X Zhang Y-H Li J Flavonoids inhibit cell proliferation and induce apoptosis and autophagy through downregulation of PI3Kγ mediated PI3K/AKT/mTOR/p70S6K/ULK signaling pathway in human breast cancer cells. *Sci Rep.* (2018) 8:11255. 10.1038/s41598-018-29308-7 30050147 PMC6062549

[31] Liu Z Ding D Cui H Wang C Liu J Chen L A study on the volatile compounds in *Elaeagnus angustifolia* L. flowers during flowering season by gas chromatography-mass spectrometry coupled with advanced chemometrics. *J Food Qual.* (2021) 2021:1–8. 10.1155/2021/7111120

[32] Hanahan D Weinberg RA. The hallmarks of cancer. *Cell.* (2000) 100:57–70. 10.1016/s0092-8674(00)81683-9 10647931

[33] Plati J Bucur O Khosravi-Far R. Dysregulation of apoptotic signaling in cancer: molecular mechanisms and therapeutic opportunities. *J Cell Biochem.* (2008) 104:1124–49. 10.1002/jcb.21707 18459149 PMC2941905

[34] Hemann MT Lowe SW. The p53-Bcl-2 connection. *Cell Death Differ.* (2006) 13:1256–9. 10.1038/sj.cdd.4401962 16710363 PMC4590992

[35] Fridman JS Lowe SW. Control of apoptosis by p53. *Oncogene.* (2003) 22:9030–40. 10.1038/sj.onc.1207116 14663481

[36] Yu H Lee H Herrmann A Buettner R Jove R. Revisiting STAT3 signalling in cancer: new and unexpected biological functions. *Nat Rev Cancer.* (2014) 14:736–46. 10.1038/nrc3818 25342631

[37] Guanizo AC Fernando CD Garama DJ Gough DJ. STAT3: a multifaceted oncoprotein. *Growth Factors.* (2018) 36:1–14. 10.1080/08977194.2018.1473393 29873274

[38] Sirkisoon SR Carpenter RL Rimkus T Anderson A Harrison A Lange AM Interaction between STAT3 and GLI1/tGLI1 oncogenic transcription factors promotes the aggressiveness of triple-negative breast cancers and HER2-enriched breast cancer. *Oncogene.* (2018) 37:2502–14. 10.1038/s41388-018-0132-4 29449694 PMC5948110

[39] Lee H Jeong AJ Ye S-K. Highlighted STAT3 as a potential drug target for cancer therapy. *BMB Rep.* (2019) 52:415–23. 10.5483/BMBRep.2019.52.7.152 31186087 PMC6675244

[40] Darnell JE Jr. STATs and gene regulation. *Science.* (1997) 277:1630–5. 10.1126/science.277.5332.1630 9287210

[41] Kamran MZ Patil P Gude RP. Role of STAT3 in cancer metastasis and translational advances. *Biomed Res Int.* (2013) 2013:421821. 10.1155/2013/421821 24199193 PMC3807846

[42] Al Zaid Siddiquee K Turkson J. STAT3 as a target for inducing apoptosis in solid and hematological tumors. *Cell Res.* (2008) 18:254–67. 10.1038/cr.2008.18 18227858 PMC2610254

[43] Chun J Song K Kim YS. Sesquiterpene lactones-enriched fraction of *Inula helenium* L. induces apoptosis through inhibition of signal transducers and activators of transcription 3 signaling pathway in MDA-MB-231 breast cancer cells. *Phytother Res.* (2018) 32:2501–9. 10.1002/ptr.6189 30251272

[44] Romeo MA Gilardini Montani MS Benedetti R Santarelli R D’Orazi G Cirone M. STAT3 and mutp53 engage a positive feedback loop involving HSP90 and the mevalonate pathway. *Front Oncol.* (2020) 10:1102. 10.3389/fonc.2020.01102 32754441 PMC7367154

[45] Pham T-H Park H-M Kim J Hong JT Yoon D-Y. STAT3 and p53: dual target for cancer therapy. *Biomedicines.* (2020) 8:637. 10.3390/biomedicines8120637 33371351 PMC7767392

[46] Niu G Wright KL Ma Y Wright GM Huang M Irby R Role of Stat3 in regulating p53 expression and function. *Mol Cell Biol.* (2005) 25:7432–40. 10.1128/mcb.25.17.7432-7440.2005 16107692 PMC1190305

[47] Lin J Tang H Jin X Jia G Hsieh J-T. p53 regulates Stat3 phosphorylation and DNA binding activity in human prostate cancer cells expressing constitutively active Stat3. *Oncogene.* (2002) 21:3082–8. 10.1038/sj.onc.1205426 12082540

[48] Tan FH Putoczki TL Stylli SS Luwor RB. The role of STAT3 signaling in mediating tumor resistance to cancer therapy. *Curr Drug Targets.* (2014) 15:1341–53. 10.2174/1389450115666141120104146 25410411

[49] Hientz K Mohr A Bhakta-Guha D Efferth T. The role of p53 in cancer drug resistance and targeted chemotherapy. *Oncotarget.* (2017) 8:8921–46. 10.18632/oncotarget.13475 27888811 PMC5352454

